# Continuous-variable geometric phase and its manipulation for quantum computation in a superconducting circuit

**DOI:** 10.1038/s41467-017-01156-5

**Published:** 2017-10-20

**Authors:** Chao Song, Shi-Biao Zheng, Pengfei Zhang, Kai Xu, Libo Zhang, Qiujiang Guo, Wuxin Liu, Da Xu, Hui Deng, Keqiang Huang, Dongning Zheng, Xiaobo Zhu, H. Wang

**Affiliations:** 1 Department of Physics, Zhejiang University, Hangzhou, Zhejiang 310027 China; 2 Fujian Key Laboratory of Quantum Information and Quantum Optics, College of Physics and Information Engineering, Fuzhou University, Fuzhou, Fujian 350116 China; 3 Institute of Physics, Chinese Academy of Sciences, Beijing, 100190 China; 4 Synergetic Innovation Center of Quantum Information and Quantum Physics, University of Science and Technology of China, Hefei, Anhui 230026 China; 5 School of Physical Sciences, University of Chinese Academy of Sciences, Beijing, 100049 China

## Abstract

Geometric phase, associated with holonomy transformation in quantum state space, is an important quantum-mechanical effect. Besides fundamental interest, this effect has practical applications, among which geometric quantum computation is a paradigm, where quantum logic operations are realized through geometric phase manipulation that has some intrinsic noise-resilient advantages and may enable simplified implementation of multi-qubit gates compared to the dynamical approach. Here we report observation of a continuous-variable geometric phase and demonstrate a quantum gate protocol based on this phase in a superconducting circuit, where five qubits are controllably coupled to a resonator. Our geometric approach allows for one-step implementation of *n*-qubit controlled-phase gates, which represents a remarkable advantage compared to gate decomposition methods, where the number of required steps dramatically increases with *n*. Following this approach, we realize these gates with *n* up to 4, verifying the high efficiency of this geometric manipulation for quantum computation.

## Abstract (web summary)

Geometric phase is of fundamental interest and has practical application in quantum computation. Here the authors observe continuous-variable geometric phase in a superconducting circuit and demonstrate a multi-qubit controlled phase gate protocol based on this geometric effect.

## Introduction

A quantum system, when undergoing a cyclic evolution in the quantum state space, will acquire a geometric phase that is determined by the path traversed by the system^1, 2^. This geometric effect has close relations with a variety of physical phenomena in areas, including optics, molecular physics, quantum field theories, and condensed matter physics^3^. Unlike the time- and energy-dependent dynamical phase, geometric phase depends only on the global property of the evolution path, e.g., the enclosed area, and is not affected by any deformation of the path that preserves the enclosed area. As such, geometric phase is robust against certain types of noise perturbations and can be used for coherent manipulation of quantum states and for implementation of quantum logic gates^4, 5^. The behaviors of geometric phases subject to different noise sources have been investigated for both the adiabatic and nonadiabatic evolutions. Previous theoretical^6^ and experimental^7, 8^ results demonstrated the robustness of adiabatic geometric phase (Berry phase)^1^ against random fluctuations of classical control parameters. In addition, it has been shown that the geometric phases in certain systems are insensitive to decoherence effects arising from coupling to reservoirs^9, 10^.

So far, Berry’s phase and its extensions in various discrete-variable systems, e.g., qubits, have been experimentally investigated^7, 8, 11–14^ and used for realization of elementary quantum gates^5, 15–18^. Geometric phases of continuous-variable systems, or harmonic oscillators, whose states are defined in an infinite-dimensional Hilbert space, are also useful for quantum gate operations. In the context of ion-trap architectures, a harmonic vibrational mode has been utilized for implementing high-fidelity quantum gates for ionic qubit^4^. Superconducting circuit quantum electrodynamics (QED) systems represent another scalable platform for quantum information processing^19^. In a recent experiment^20^, the adiabatic geometric phase of the quantized electromagnetic field stored in a resonator was measured in a circuit QED device, where the resonator was dispersively coupled to a qubit and driven by a microwave pulse whose amplitude and phase were slowly and cyclically changed. The geometric phase was calculated as the difference between the total phase measured for the area-enclosed path of the resonator state in phase space and that for a straight line path, the latter of which produced the same dynamical phase but no geometric one. More recently, a similar resonator-induced phase was used to realize two-qubit gates in a three-dimensional circuit QED architecture^21, 22^, where four transmon qubits with fixed frequencies were dispersively coupled to a cavity. To cancel the effects of unwanted interactions, a refocused gate scheme was designed, where the cavity was sequentially driven by 8 pulses, intervened by suitably arranged *π* pulses applied to the qubits.

Here we report on the observation of the geometric phase of an electromagnetic resonator in a superconducting circuit QED system, based on which we demonstrate a universal protocol for realizing multi-qubit controlled-phase gates in one step. In our experiment, the state of the resonator is nonadiabatically displaced with a constant-amplitude microwave drive along a circuit in phase space conditional on the state of the qubit coupled to the resonator, and the geometric phase associated with this cyclic evolution is measured by the qubit’s Ramsey interference experiment. Using this phase, we realize the two-qubit controlled-phase (CZ) gate, the three-qubit controlled–controlled-phase (CCZ) gate—the equivalent of the Toffoli gate under a change of the target basis, and the four-qubit controlled–controlled–controlled-phase (CCCZ) gate. The geometric CZ gate is calibrated by quantum process tomography (QPT) and randomized benchmarking (RB), each giving a fidelity of about 0.94; the CCZ and CCCZ gates, both achieved without resorting to the two-qubit-gate decomposition, yield the QPT fidelities of 0.868 ± 0.004 and 0.817 ± 0.006, respectively, which compare favorably to the results obtained by step-by-step dynamical approaches^23–28^. Taking advantage of the qubit tunability in our setup, we implement these resonator-induced phase gates with a single pulse driving the resonator, which is different from the long pulse sequence used in the experiment of ref. ^22^. Our scheme also minimizes the wiring complexity, i.e., with a bus resonator we can achieve noise-resilient geometric entangling gates^10^ among arbitrarily chosen qubits. Further numerical simulations suggest that, with optimal circuit designs, the two-qubit CZ gate fidelity can be raised to above the surface code threshold for fault tolerance^29, 30^, while the multi-qubit controlled-phase gates, directly applicable in the quantum search algorithm^31^ and quantum error correction, can be executed in one step and with high fidelity.

## Results

### Device and geometric phase

**Fig. 1:**
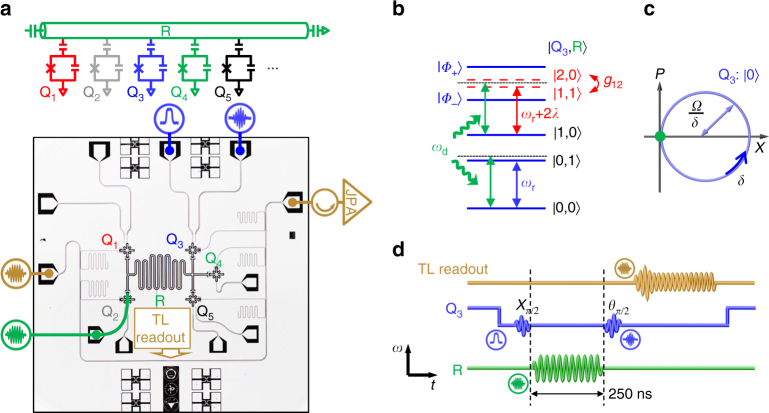
Device and scheme for measuring geometric phase. **a** Device schematic and image illustrating the five frequency-tunable qubits, labeled from Q_1_ to Q_5_, and the bus resonator R, which has a fixed bare frequency (resonator frequency in absence of qubits) *ω*_rb_/2*π* ≈ 5.585 GHz. The color-coded icons identify the pads where pulses are injected onto the circuit chip. The transmission line (TL) carries the multi-tone microwave pulse through the circuit chip, which is amplified by a Josephson parametric amplifier (JPA) at low temperature and then demodulated at room temperature to yield the state of all qubits. **b** Energy level configuration of the qubit-resonator system. The strong coupling between $\left| {2,0} \right\rangle$ and $\left| {1,1} \right\rangle$ produces the dressed states $\left| {{\phi _ \pm }} \right\rangle$ whose energy levels are well separated. A microwave drive with a tone of *ω*_d_ that is slightly detuned from *ω*_r_ by *δ* can or cannot excite the resonator depending on whether the qubit is in the state $\left| 0 \right\rangle$ or $\left| 1 \right\rangle$. **c** Resonator’s phase-space displacement conditional on the qubit state $\left| 0 \right\rangle$. In the drive frame, the resonator, initially in its ground state, is displaced by the microwave drive of an amplitude *Ω* along a circle in phase space with the radium *Ω* */δ* and the angular velocity *δ* conditional on the qubit state $\left| 0 \right\rangle$. At time *T* = 2*π*/*δ*, the resonator makes a cyclic evolution, returning to the ground state, but acquires a conditional geometric phase proportional to the enclosed phase-space area. **d** Ramsey interference sequence plotted in the frequency vs. time plane. The geometric operation, resulting from the combination of the microwave drive (green sinusoid) and the qubit-resonator coupling, is sandwiched in between the two *π*/2 rotations (blue sinusoids with Gaussian envelopes), X_*π*/2_ and *θ*_*π*/2_, whose rotation axes are in the *xy* plane of the Bloch sphere and differ by an angle of *θ*. The corresponding geometric phase *β* is revealed by measuring the qubit $\left| 1 \right\rangle$-state probability as a function of *θ*, using the microwave pulse through the TL readout line (light brown sinusoid with a ring-down shape at the beginning)

Our circuit QED architecture consists of five frequency-tunable superconducting Xmon qubits, labeled from Q_1_ to Q_5_, all coupled to a bus resonator R (see Fig. 1a and “Methods” section). First, we introduce the single-qubit experiment for observing the resonator’s geometric phase, measured through Q_3_’s Ramsey interference. The qubit-resonator (Q_3_-R) level configuration is illustrated in Fig. 1b, where *c* and *d* in the joint state $\left| {c,d} \right\rangle$ denote the excitation numbers of the qubit and the resonator, respectively. The qubit $\left| 0 \right\rangle \leftrightarrow \left| 1 \right\rangle$ transition at the tone *ω*_01_ is coupled to the resonator with a coupling strength *g*_01_/2*π* = 20.1 MHz. When the qubit-resonator detuning *Δ* (≡ *ω*_01_ − *ω*_rb_) is much larger than *g*_01_ so that the energy levels $\left| {1,0} \right\rangle$ and $\left| {0,1} \right\rangle$ are well separated as illustrated in Fig. 1b, there is no population exchange between these two levels; the dispersive coupling results in a qubit-state-dependent resonator frequency shift, described by the effective Hamiltonian $\hbar \lambda \left( {\left| 1 \right\rangle \left\langle 1 \right| - \left| 0 \right\rangle \left\langle 0 \right|} \right){a^\dag }a$, where *a* ^†^ and *a* are the creation and annihilation operators for the photons stored in the resonator, $\hbar$ is the Planck constant, and $\lambda = g_{01}^2{\rm{/}}\varDelta$. We note that this effective Hamiltonian does not include the coupling of the qubit transition $\left| 1 \right\rangle$ ↔ $\left| 2 \right\rangle$ and the resonator, which is quasi-resonant (see below). The resonator is off-resonantly driven by an external microwave field with the amplitude *Ω* and the tone *ω*_d_. When the qubit is initially in the state $\left| 0 \right\rangle$, it remains in this state, and the effective Hamiltonian for the driven resonator, in the frame rotating at *ω*_d_ (the drive frame) becomes

1 $$H = - \hbar \delta {a^\dag }a + \hbar {\varOmega }\left( {a + {a^{\rm{\dag }}}} \right),$$

where *δ* = *ω*_d_ − *ω*_r_ and *ω*_r_ (≡ *ω*_rb_ − *λ*) denotes the resonator frequency conditional on the qubit state $\left| 0 \right\rangle$.

With the Hamiltonian shown in Eq. (1), the resonator evolves from the ground state to the coherent state $\left| {\phi (t)} \right\rangle = {\mathrm{e}^{i\beta (t)}}\left| {\alpha (t)} \right\rangle$, where $\beta (t) = - \frac{{{\Omega ^2}}}{\delta }\left[ {t - \frac{1}{\delta }{\rm{sin}}\left( {\delta t} \right)} \right]$, and $\alpha (t) = \frac{\Omega }{\delta }\left( {1 - {\mathrm{e}^{i\delta t}}} \right)$ is the complex amplitude of the coherent field. After a time *T* = 2*π*/*δ*, the resonator makes a cyclic evolution, returning to the initial state but acquiring a phase, *β* = −2*π*(*Ω*/*δ*)^2^. The total phase *β* is best visualized in phase space spanned by the two quadratures *X* = (*a* + *a* ^†^)/2 and *P* = (*a* − *a* ^†^)/2*i*, where the resonator state moves around a circle with the radium *Ω*/*δ* and angular velocity *δ*, as shown in Fig. 1c; *β* is proportional to the enclosed phase-space area^4^. We note that the acquired phase contains no dynamical contribution, defined as^2^ $- \frac{1}{\hbar }{\int}_0^T \left\langle H \right\rangle {\rm{d}}t$, in the drive frame, while it has both the Aharonov–Anandan geometric contribution and the aforementioned dynamical component when it is viewed in the interaction frame^32^, i.e., the frame rotating at the resonator frequency, but where it is still proportional to the enclosed phase-space area^4^. As such, for the cyclic evolution of a continuous-variable system, the phase that depends on the enclosed phase-space area in the interaction frame or in the drive frame, other than the Aharonov–Anandan phase, is usually termed as the geometric phase^4, 20^, and the area-independent part corresponds to the dynamical component. We further note that the acquired phase is insensitive to the resonator dissipation, as shown elsewhere^10^.

The strong coupling between the qubit-resonator states $\left| {1,1} \right\rangle$ and $\left| {2,0} \right\rangle$ is used to freeze the resonator’s evolution associated with the qubit state $\left| 1 \right\rangle$. When these two states are on near-resonance, they are strongly coupled and form two dressed states $\left| {{\phi _ \pm }} \right\rangle$ with modified energy levels that are separated by about *g*_12_ (see Supplementary Note 1), where *g*_12_ $\left( { \approx \sqrt 2 {g_{01}}} \right)$ is the coupling strength between the qubit $\left| 1 \right\rangle$ ↔ $\left| 2 \right\rangle$ transition and the resonator (Fig. 1b). Under the weak driving condition $\Omega \ll {g_{12}}$, the external field cannot drive the system to evolve from the state $\left| {1,0} \right\rangle$ to either one of $\left| {{\phi _ \pm }} \right\rangle$, but shifts its energy level and produces a dynamical phase. We eliminate this dynamical phase by adjusting the qubit-resonator detuning so that the energy shifts associated with the off-resonant couplings to $\left| {{\phi _ \pm }} \right\rangle$ cancel each other. Under this condition, nothing changes when the qubit is in $\left| 1 \right\rangle$ (see detailed calculations in Supplementary Note 1). The geometric phase acquired by the resonator can be encoded in the relative probability amplitude of the qubit basis states $\left| 0 \right\rangle$ and $\left| 1 \right\rangle$, and measured in a Ramsey interference experiment.

**Fig. 2:**
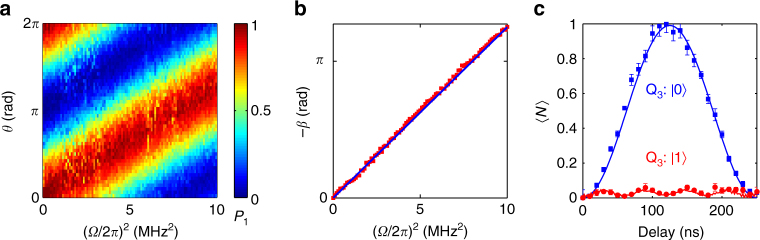
Ramsey interference for geometric phase. **a** Occupation probability *P*_1_ of Q_3_ in $\left| 1 \right\rangle$ as a function of *θ* and *Ω* ^2^, which is measured using the pulse sequence shown in Fig. 1d with the drive detuning *δ*/2*π* = 4 MHz. **b** −*β* vs. *Ω* ^2^ (red dots), where *β* is obtained by tracing the *P*_1_-maximum contour in **a**: For each Ramsey trace of *P*_1_ vs. *θ* sliced along a fixed *Ω* ^2^, we perform the cosinusoidal fit with the phase offset giving the value of *β*. The blue solid line shows the theoretical result. **c** Measured average photon numbers with error bars of the resonator as functions of time during the application of the microwave drive with *Ω*/2*π* = 2 MHz conditional on the qubit states $\left| 0 \right\rangle$ (blue dots) and $\left| 1 \right\rangle$ (red dots). Error bars represent statistical errors (s.d.) of repeated sets of measurement. Lines are the numerical results

During the application of the resonator drive, the $\left| 0 \right\rangle \leftrightarrow \left| 1 \right\rangle$ and $\left| 1 \right\rangle \leftrightarrow \left| 2 \right\rangle$ transitions of Q_3_ are blue-detuned from the resonator frequency *ω*_r_/2*π* by 284 and 39 MHz, respectively. The resulting geometric phase is observed by the Ramsey-type measurement, where the above-mentioned geometric operation is sandwiched in between two *π*/2 rotations on Q_3_ as illustrated in Fig. 1d (also see “Methods” section). In Fig. 2a, we present the measured probability of Q_3_ in $\left| 1 \right\rangle$ after the second *π*/2 rotation, *P*_1_, as a function of *θ* and *Ω* ^2^ in a two-dimensional colourmap, where *Ω* is calibrated by measuring the drive-generated resonator photon number with Q_4_. Tracing the contour of the *P*_1_-maximum yields, the linear dependence of the negative geometric phase, −*β*, on *Ω* ^2^, which agrees exceptionally well with the analytic solution (solid line in Fig. 2b). Figure 2c displays the average photon numbers with error bars of the resonator as functions of time during application of the drive with Q_3_ in $\left| 0 \right\rangle$ (blue) and $\left| 1 \right\rangle$ (red), which are measured by tuning Q_4_, initially in its ground state, on resonance with the resonator for an interaction time before its readout; the resulting *P*_1_ vs. time curve is used to extract the photon populations. As expected, when Q_3_ is in the state $\left| 1 \right\rangle$, the resonator almost remains unpopulated; for the qubit state $\left| 0 \right\rangle$, the resonator makes a cyclic evolution, returning to the ground state after the duration *T* = 250 ns.

### Geometric two-qubit CZ gate

Now we turn to the implementation of the geometric CZ gate with Q_1_ and Q_5_. We arrange the $\left| 0 \right\rangle$ ↔ $\left| 1 \right\rangle$ transition frequencies of Q_1_ and Q_5_ to be blue-detuned from the resonator frequency *ω*_r_/2*π* by ~264 and 285 MHz, respectively, where the qubit lifetimes are measured to be around 14.8 μs for Q_1_ and 12.3 μs for Q_5_, and the Gaussian dephasing times^33^ $T_2^ *$ of both qubits are around 5 μs. With this arrangement and the qubit anharmonicities (see Supplementary Note 2), the $\left| 1 \right\rangle$ ↔ $\left| 2 \right\rangle$ transition frequencies of Q_1_ and Q_5_ are blue-detuned from *ω*_r_/2*π* by ~19 and 41 MHz, respectively, which are comparable to the coupling strength *g*_12_/2*π* of $\sqrt 2 \times 20.9$ MHz for Q_1_ and $\sqrt 2 \times 19.8$ MHz for Q_5_, i.e., the $\left| 1 \right\rangle \leftrightarrow \left| 2 \right\rangle$ transitions of both qubits are on near-resonance with the resonator. The detuning between Q_1_ and Q_5_, 22 MHz, is much larger than the dispersive coupling strengths between the $\left| 0 \right\rangle \leftrightarrow \left| 1 \right\rangle$ transitions of both qubits to minimize the resonator-induced qubit excitation exchange. With these settings and in the drive frame, the external microwave field will drive the resonator to traverse a circle in phase space when both qubits are in the state $\left| 0 \right\rangle$; when one qubit is in $\left| 1 \right\rangle$, the strong coupling between the joint states $\left| {1,1} \right\rangle$ and $\left| {2,0} \right\rangle$ of this qubit and the resonator is again used to freeze the resonator’s evolution for the same reason outlined in the single-qubit experiment, and so is the case when both qubits are in $\left| 1 \right\rangle$ (see Supplementary Note 1). A geometric two-qubit phase gate can thus be constructed, where a geometric phase *β* is produced if and only if both qubits are in the state $\left| 0 \right\rangle$.

To examine the phase acquired by each of the two-qubit computational states during the gate operation, we perform the Ramsey-type measurements on each qubit with the other qubit in $\left| 0 \right\rangle$ and $\left| 1 \right\rangle$, respectively (see Supplementary Fig. 3 and Supplementary Note 3). In addition to the dominant *Ω* ^2^-dependent geometric phase *β* gained by $\left| {0,0} \right\rangle$, the Ramsey data show that the two-qubit computational states also accumulate different but small dynamical phases, which constitute the majority of phase errors to the CZ gate in our experimental realization. We perform additional single-qubit rotations to partially compensate for the dynamical-phase-induced errors.

**Fig. 3:**
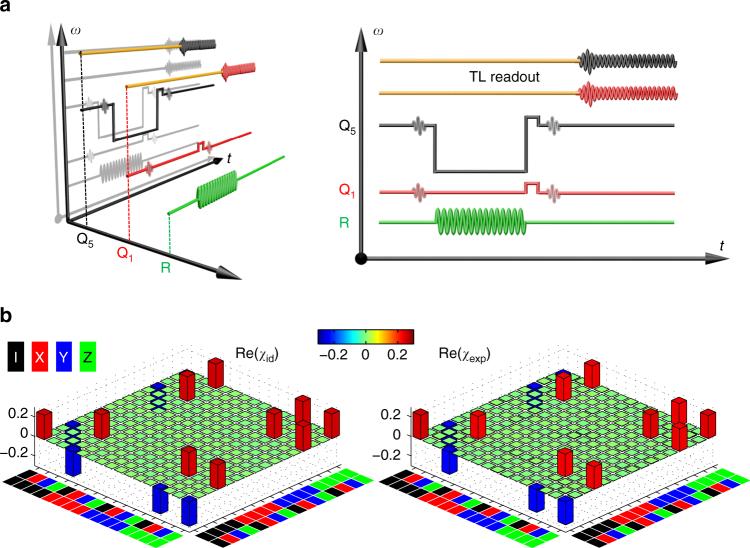
QPT of the geometric two-qubit CZ gate, obtained with the drive amplitude $\Omega {\rm{/}}2\pi \approx \sqrt {7.0}$ MHz and detuning *δ*/2*π* = 4 MHz. **a** Pulse sequences illustrated in three dimensions (left) and projected to two dimensions (right), with the axes as labeled. For each qubit, the first sinusoid with a Gaussian envelope is for state preparation, which is varied to generate one of the four states $\left\{ {\left| 0 \right\rangle } \right.$, $\left( {\left| 0 \right\rangle - i\left| 1 \right\rangle } \right){\rm{/}}\sqrt 2$, $\left( {\left| 0 \right\rangle + \left| 1 \right\rangle } \right){\rm{/}}\sqrt 2$, $\left. {\left| 1 \right\rangle } \right\}$}; the second sinusoid with a Gaussian envelope is also variable, acting as the rotation pulse needed in QST; sandwiched in between the two sinusoids is the big square pulse used to adjust the qubit energy levels of Q_5_ (there is no frequency adjustment on Q_1_), which combines with the resonator microwave drive to fulfill the CZ gate; the next small square pulse produces a single-qubit rotation on each qubit to partially compensate for the dynamical phase accumulated during the CZ gate; finally qubits are measured by demodulation of the two-tone microwave through the TL readout line (light brown lines with color-coded sinusoids). Here the readout and gate frequencies of Q_5_ are different for minimizing the Q_1_–Q_5_ interaction during readout. **b** Ideal (*χ*_id_, left) and experimental (*χ*_exp_, right) quantum process matrices. The color code for Pauli basis {I, X, Y, Z} is shown at the top-left corner. Imaginary components of *χ*_exp_ are measured to be no larger than 0.015 in magnitude. *χ*_exp_ has a fidelity *F* = Tr(*χ*_id_ *χ*_exp_) = 0.936 ± 0.013. The $\left| 2 \right\rangle$-state occupation probability of each qubit averaged over the 16 output states is no higher than 0.015 in a separate measurement. We also perform the CZ gate with Q_1_ and Q_3_, and obtain a similar gate fidelity

To characterize the resulting CZ gate, the two-qubit QPT is performed by creating 16 distinct two-qubit input states and mapping out these input and corresponding output states with quantum state tomography (QST), using the pulse sequence illustrated in Fig. 3a. The resulting experimental process matrix *χ*_exp_ is shown in Fig. 3b together with the ideal matrix *χ*_id_ for comparison, which corresponds to a gate fidelity of 0.936 ± 0.013. We also examine the gate performance using interleaved RB, where we insert the CZ gate between random gates from the one- and two-qubit Clifford groups, measuring a fidelity of 0.939±0.011 (see Supplementary Fig. 4 and Supplementary Note 3). The Bell state produced by this gate has a fidelity of 0.949 ± 0.018 and a concurrence of 0.914 ± 0.038.

The experimental CZ fidelity values agree well with the numerical simulation using the Lindblad master equation, where the pure dephasing times *T*_Φ_ are set to be around 15 μs for both qubits. Empirically we have found^33^ that using the Markovian *T*_Φ_ much longer than the Gaussian $T_2^ *$ ensures a good agreement between the theory and experiment for sequences much shorter than $T_2^ *$.

### Geometric three-qubit CCZ gate

One important feature of our geometric approach is that it allows one-step implementation of an *n*-qubit controlled-phase gate—the key element in the quantum search algorithm^31^ and quantum error correction, irrespective of *n*, which is in remarkable contrast with methods based on gate decomposition, where the number of required two-qubit gates increases dramatically with *n* ^34^. Here we demonstrate the three-qubit CCZ gate, which produces a *π*-phase shift if and only if all three qubits are in $\left| 0 \right\rangle$, without using concatenated two-qubit gates as required in previous experiments^23–28^. The CCZ gate, in combination with single-qubit rotations, is equivalent to the Toffoli gate that inverts the state of the target qubit conditional on the state of the two control qubits, and which is essential for constructing a universal set of quantum operations^35^ and for quantum error correction^24^.

**Fig. 4:**
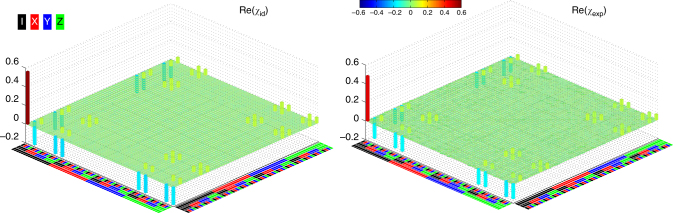
QPT of the geometric three-qubit CCZ gate, obtained with the drive amplitude $\Omega {\rm{/}}2\pi \approx \sqrt {7.5}$ MHz and detuning *δ*/2*π* = 4 MHz. The color code for Pauli basis {I, X, Y, Z} is shown at the top-left corner. The process matrix is reconstructed by preparing a complete set of 64 input states, and measuring both the input and output density matrices using QST. The ideal (*χ*_id_) and experimental (*χ*_exp_) quantum process matrices are presented in the left and right panels, respectively. Imaginary components of *χ*_exp_ are measured to be no larger than 0.063 in magnitude. The fidelity of *χ*_exp_ is 0.868 ± 0.004. The $\left| 2 \right\rangle$-state occupation probability of each qubit resulting from the drive *Ω* is no higher than 0.025 in a separate measurement, in which the test qubit is initialized in $\left| 1 \right\rangle$ and the other two qubits are in $\left| 0 \right\rangle$

We realize the CCZ gate with Q_1_, Q_3_, and Q_5_ by carefully adjusting the qubit level configuration (see Supplementary Note 4): The $\left| 0 \right\rangle$ ↔ $\left| 1 \right\rangle$ transition frequencies of Q_1_, Q_3_, and Q_5_ are blue-detuned from the resonator frequency *ω*_r_/2*π* by ~268, 249, and 285 MHz, respectively, and the $\left| 1 \right\rangle$ ↔ $\left| 2 \right\rangle$ transition frequencies are blue-detuned from *ω*_r_/2*π* by ~23, 4, and 41 MHz. At the above-mentioned frequencies the qubit lifetimes are around 14.8, 16.4, and 12.3 μs. The reconstructed experimental QPT matrix *χ*_exp_ has a fidelity of 0.868 ± 0.004 (Fig. 4), which agrees well with the Lindblad master equation simulation using *T*_Φ_ ≈ 10 μs for all three qubits. The slight drop of *T*_Φ_, which is still much longer than $T_2^ *$, suggests that other error sources may be involved in the three-qubit implementation, which will be investigated next. The Ramsey interference patterns of each of the three qubits conditional on the state of the rest two qubits are shown in Supplementary Fig. 5 with details described in Supplementary Note 4.

### Geometric four-qubit CCCZ gate

For illustration of the remarkable scaling performance of our protocol, here we implement the four-qubit CCCZ gate, which produces a *π*-phase shift if and only if all four qubits are in $\left| 0 \right\rangle$. An equivalent of the CCCZ gate up to single-qubit rotations was recently implemented with trapped ions for the first time, which requires 11 two-qubit gates and has a fidelity of 0.705 ± 0.003 as characterized by a limited tomography procedure^28^. It was also reported with the same setup^28^ that the three-qubit Toffoli gate requires 5 two-qubit gates and has a fidelity of 0.896 ± 0.002.

**Fig. 5:**
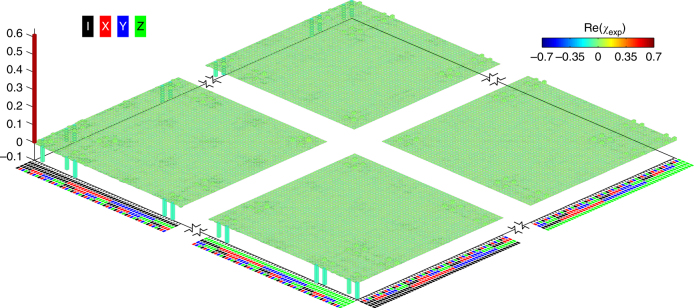
QPT of the geometric four-qubit CCCZ gate, obtained with the drive amplitude $\Omega {\rm{/}}2\pi \approx \sqrt {6.9}$ MHz and detuning *δ*/2*π* = 4 MHz. The color code for Pauli basis {I, X, Y, Z} is shown at the top-left corner. The partially shown process matrix *χ*_exp_ is reconstructed by preparing a complete set of 256 input states, and measuring both the input and output density matrices using QST. Imaginary components of *χ*_exp_ are measured to be no larger than 0.062 in magnitude. The fidelity of *χ*_exp_ is 0.817 ± 0.006. Numerical simulation suggests that the $\left| 2 \right\rangle$-state occupation probability for each qubit is no higher than 0.025

Our four-qubit CCCZ gate is implemented on the same device but in a separate cooldown, and therefore the device parameters might drift very slightly. We realize the CCCZ gate with Q_1_, Q_2_, Q_4_, and Q_5_ by carefully adjusting the qubit level configuration, so that the $\left| 0 \right\rangle$ ↔ $\left| 1 \right\rangle$ transition frequencies are blue-detuned from the resonator frequency *ω*_r_/2*π* by ~270, 247, 282, and 262 MHz, respectively, and the $\left| 1 \right\rangle$ ↔ $\left| 2 \right\rangle$ transition frequencies are blue-detuned from *ω*_r_/2*π* by ~25, 5, 39, and 18 MHz. At the above-mentioned frequencies, the qubit lifetimes are around 16.5, 13.5, 15.4, and 13.9 μs for Q_1_, Q_2_, Q_4_, and Q_5_, respectively, and the Gaussian dephasing times of all qubits are measured to be around 4 μs. For the CCCZ gate, we drive the resonator through Q_3_’s microwave line. The reconstructed experimental QPT matrix *χ*_exp_ involves 256 input states and 256 output states, and has a fidelity of 0.817 ± 0.006 (Fig. 5), which is close to the numerical simulation taking *T*_Φ_ to be close to 10 μs for all qubits. Different from the two- and three-qubit experiments, here right before the tomographic pulses to characterize the output states, we do not append single-qubit rotations to partially compensate for the dynamical-phase-induced errors, instead we add the desired correction phase to each qubit’s tomographic pulses following the procedure used in refs. ^29, 30^. The much less drop in fidelities from the CCZ gate to the CCCZ gate in our case as compared with the very recent ion-trap experiment^28^ verifies the remarkable scaling performance of our multi-qubit controlled-phase gate protocol.

## Discussion

The dynamical effect, one of the main error sources in the current multi-qubit controlled-phase gate implementations, can be suppressed with the quantum-bus circuit architecture (Fig. 1a), featuring stronger qubit-resonator couplings, larger qubit anharmonicities, and larger differences in qubit anharmonicities, which would enable geometric entangling gates with significantly higher fidelity targeting two and more arbitrarily chosen qubits with our one-step scheme. As verified by numerical simulations, if the two qubits, e.g., the capacitively shunted flux qubits^36, 37^, have anharmonicities of 0.8 and 1.0 GHz, respectively, both coupled to the resonator with *g*_01_/2*π* = 38 MHz, the CZ gate fidelity can be improved to 0.991 with coherence times around 100 μs (the decoherence-free gate fidelity is 0.996), which is above the surface code threshold for fault tolerance^29, 30^; introducing a third qubit with an anharmonicities of 0.9 GHz would give a CCZ gate fidelity of 0.987 (0.994 without decoherence). The geometric gates are robust against variations of certain device parameters likely due to the imperfection of the circuit design and fabrication process, e.g., a 10% variation of g_01 _from qubit to qubit only causes the gate fidelity to vary around 10^–3^, provided that one can fine-tune each qubit’s frequency and the microwave drive parameters for an optimal gate fidelity. Using qubits with sufficiently large ratios of the anharmonicities to the qubit-resonator couplings, the geometric gates can be produced by strongly driving the qubits^38^; within this scenario, the gate speed and thus fidelity can be further significantly improved.

## Methods

### Experimental device

Our circuit QED architecture consists of five frequency-tunable superconducting Xmon qubits^29, 30^, all coupled to a bus resonator with a fixed bare frequency; each qubit can be effectively decoupled from the resonator by tuning it far off-resonant with the resonator. The qubit combinations of Q_3_, Q_1_–Q_5_ (Q_1_–Q_3_), Q_1_–Q_3_–Q_5_, and Q_1_–Q_2_–Q_4_–Q_5_ are selected in the one-, two-, three-, and four-qubit experiments, respectively, with Q_2_ serving as the microwave bridge through which the resonator can be driven and Q_4_ as the meter for measuring the resonator photon number (during the four-qubit experiment, which is done in a separate cooldown, Q_3_ serves as the microwave bridge and no qubit is used to measure the resonator photon number). Each qubit dispersively interacts with its own readout resonator, which couples to a common transmission line for multiplexed readout of all qubits. Single-shot quantum non-demolition measurement is achieved with an impedance-transformed Josephson parametric amplifier whose bandwidth is above 200 MHz at desired frequencies, following the design in ref. ^39^. We can simultaneously probe populations in the ground$\left| 0 \right\rangle$, the first-excited $\left| 1 \right\rangle$, and the second-excited $\left| 2 \right\rangle$ states of all qubits; the $\left| 2 \right\rangle$-state probability is measured in this work for examining the state-leakage error. The device and the measurement setup are sketched in Fig. 1a, with details described in Supplementary Fig. 1 and Supplementary Note 2.

### Ramsey-type measurement

The Ramsey interference sequence starts by applying an X_*π*/2_ gate that rotates Q_3_ around the *x*-axis on the Bloch sphere by an angle of *π*/2, transforming it from the ground state $\left| 0 \right\rangle$ to the superposition state $\left( {\left| 0 \right\rangle - i\left| 1 \right\rangle } \right){\rm{/}}\sqrt 2$, with the experimental sequence shown in Fig. 1d. Other qubits remain in $\left| 0 \right\rangle$ and are all far-detuned at their individual sweetpoint frequencies except for Q_4_, which is set 300 MHz below the resonator and will be used for reading out the resonator photon number. Then the external microwave drive *Ω* is applied, which is blue-detuned from the resonator conditional upon the qubit state $\left| 0 \right\rangle$ by *δ*/2*π* = 4 MHz. After a duration *T* = 250 ns, the qubit evolves to the state $\left( {{\mathrm{e}^{i\beta }}\left| 0 \right\rangle - i\left| 1 \right\rangle } \right){\rm{/}}\sqrt 2$, with the resonator going back to the ground state. A *θ*_*π*/2_ gate is subsequently applied to rotate Q_3_ by *π*/2 around the axis with a *θ*-angle to the *x*-axis in the *xy* plane. Finally the qubit is detected, with the probability of being measured in the state $\left| 1 \right\rangle$ given by ${P_1} = \frac{1}{2}\left[ {1 + {\rm{cos}}\left( {\beta + \theta } \right)} \right]$.

### Data availability

The data that support the findings of this study are available from the corresponding authors upon reasonable request.

## Electronic supplementary material

Supplementary Information
