# Supplementary material for: Continuous-variable geometric phase and its manipulation for quantum computation in a superconducting circuit
Source: Nat Commun. 2017 Oct 20;8:1061. doi: 10.1038/s41467-017-01156-5 (PMC5715165; doi:10.1038/s41467-017-01156-5)
Supplement: Supplementary file 1 — Supplementary Information [file 41467_2017_1156_MOESM1_ESM.pdf]

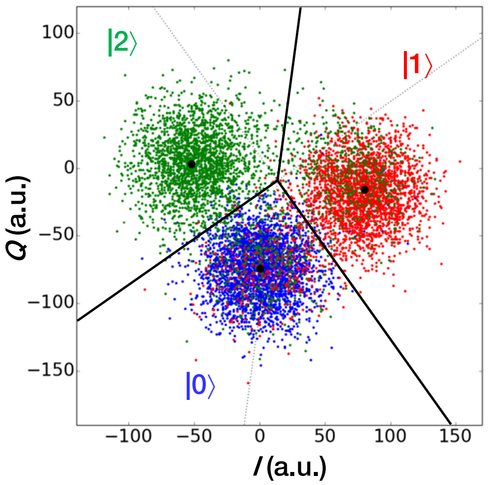

Supplementary Figure 1. **Qubit readout.** Typical microwave readout data are plotted in the I-Q plane for the $|0\rangle$, $|1\rangle$, and $|2\rangle$ states of an Xmon qubit. For the data points of the same colour, we repetitively prepare the qubit in the corresponding initial state and measure the I-Q outcomes, which are categorized into different final states according to the dividing lines. The readout pulse is 1.2 µs-long and the repetition is 3000.

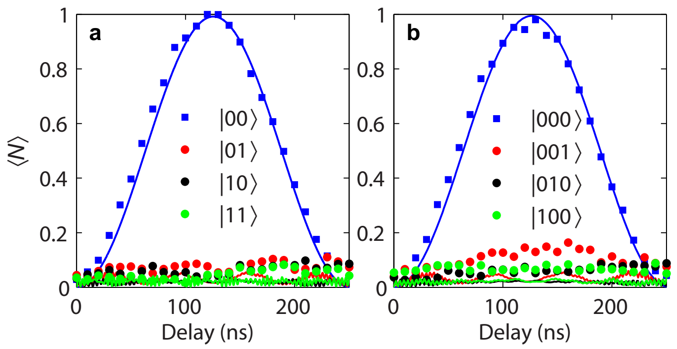

Supplementary Figure 2. **Resonator dynamics during geometric operations.** Plotted are the evolutions of the average resonator photon number during the two-qubit (**a**) and three-qubit (**b**) geometric operations with the drive amplitude $\Omega/2\pi = 2$ MHz and detuning $\delta/2\pi = 4$ MHz. The photon numbers, associated with different computational states for the two qubits of $|\mathrm{Q}_1\mathrm{Q}_5\rangle$ and the three qubits of $|\mathrm{Q}_1\mathrm{Q}_3\mathrm{Q}_5\rangle$ as labeled, are measured using Q_4. Lines are numerical simulations without considering the microwave crosstalk on the circuit chip. With the microwave crosstalk, qubits are also slightly driven when the drive is supposed to act on the resonator only.

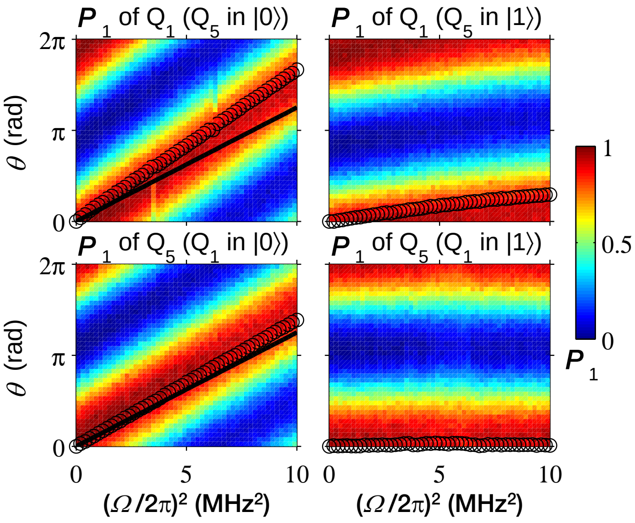

Supplementary Figure 3. **Two-qubit conditional Ramsey interference patterns.** Conditional on the control qubit being in the state $|0\rangle$ or $|1\rangle$, the Ramsey-type measurements are performed on the test qubit, where a drive with a variable amplitude Ω is applied to the resonator in between the two $\pi/2$ rotations. The panels show the measured probabilities of the test qubit in $|1\rangle$, P_1, as functions of Ω^2 and θ (the angle difference between the two $\pi/2$ rotation axes). In the upper panels, Q_5 acts as the control qubit and Q_1 as the test qubit; the situation reverses in the lower panels. The parameters except Ω are the same as those in the CZ gate experiment. Open circles trace the P_1-maximum contour: For each Ramsey trace of P_1 versus θ sliced along a fixed Ω^2, we perform the cosinusoidal fit with the phase offset giving the phase difference between the states $|1\rangle$ and $|0\rangle$ of the test qubit, which is accumulated during the application of the drive Ω (shown with open circles). Solid lines in the left two panels represent the negative geometric phases calculated as functions of Ω^2 (in the drive frame the dynamical component is zero as discussed in the main text). In the right two panels the geometric phases are expected to be zero.

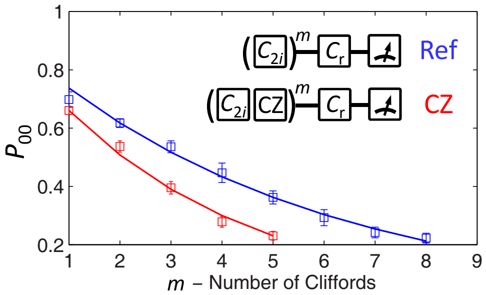

Supplementary Figure 4. **RB of the two-qubit CZ gate.** The sequence parameters as those used in Fig. 3 of the main text. The Clifford C_{2i}s are randomly chosen from the one- and two-qubit Clifford groups, the latter of which, on average, consists of 8.25 single-qubit gates and 1.5 CZ gates per Clifford. For a single-qubit gate time of 20 ns and a CZ gate time of 264 ns, the latter of which includes the extra phase gate time, the average duration of a one-qubit Clifford is 37.5 ns, and that of a two-qubit Clifford is 491 ns. C_r is the recovery gate that brings the final two-qubit state to $|00\rangle$ for a perfect sequence. Each data point with the error bar is estimated over 10 trials, and each trial is averaged over $k = 20$ random sequences. We fit the data by $P_{00} \propto p^m_{\mathrm{CZ,\ Ref}}$, and the gate fidelity is calculated as $F = 1 - 0.75\,(1 - p_{\mathrm{CZ}}/p_{\mathrm{Ref}})$.

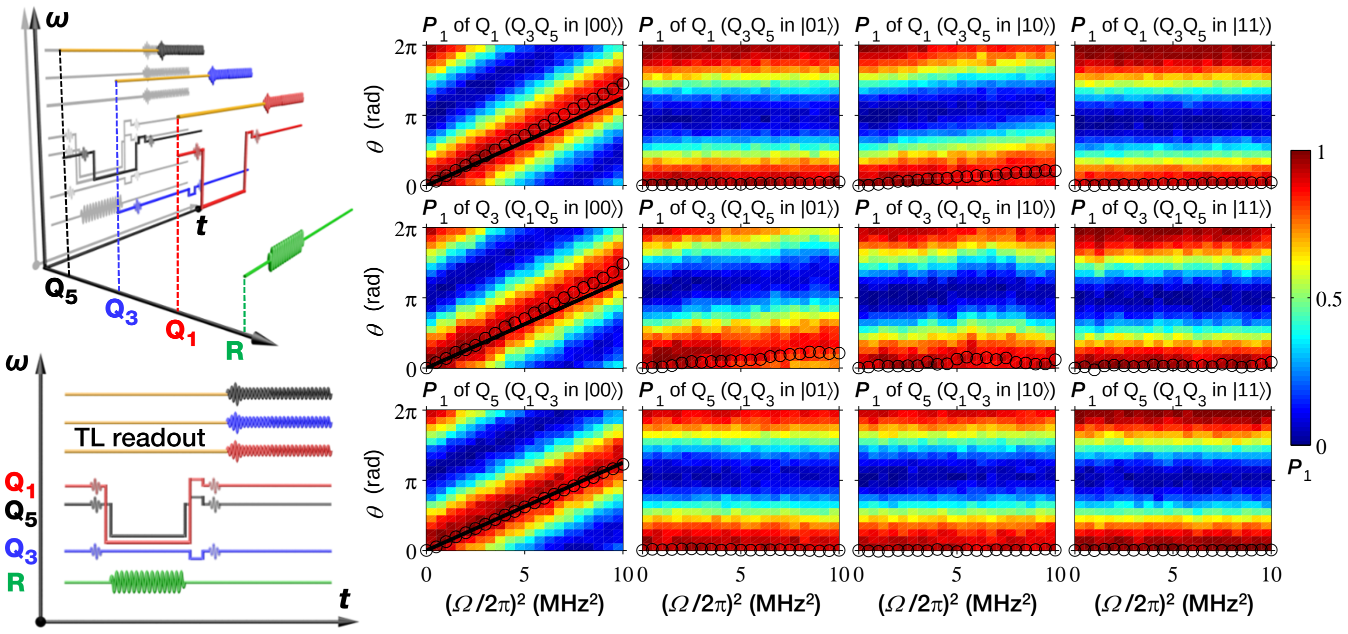

Supplementary Figure 5. **Three-qubit conditional Ramsey interference patterns.** The left panels show the pulse sequences illustrated in three dimensions and projected to two dimensions for realizing and characterizing the CCZ gate, and for performing the three-qubit conditional Ramsey-type measurements. Conditional on two control qubits being in one of the two-qubit computational states, the Ramsey-type measurements are performed on the test qubit, where a drive with a variable amplitude Ω is applied to the resonator in between the two $\pi/2$ rotations. The observed Ramsey patterns of different test qubits as functions of Ω^2 and θ are shown on the right-hand side of the figure; in the upper, middle, and lower rows, Q_1, Q_3, and Q_5 act as the test qubit, respectively. The open circles represent the measured phase difference between the states $|1\rangle$ and $|0\rangle$ of the test qubit accumulated during the application of the drive Ω. Solid lines in the Ramsey plots, if any, describe the absolute values of the calculated geometric phases as functions of Ω^2 (in the drive frame the dynamical component is zero as discussed in the main text).

	$\omega_{01}/2\pi$ (GHz)	$\omega_{\text{readout}}/2\pi$ (GHz)	$g_{\text{readout}}/2\pi$ (MHz)	T_1 (µs)	T_2^* (µs)	$g/2\pi$ (MHz)
Q_1	6.031	6.660	41	14.8	13.2	20.9
Q_2	6.036	6.719	37	6.3	3.5	20.6
Q_3	6.039	6.765	40	18.3	10.0	20.1
Q_4	6.012	6.816	37	17.2	23.8	18.8
Q_5	6.036	6.854	33	8.7	13.0	19.8
R	5.585	N/A	N/A	13.0	∞	N/A

Supplementary Table 1. **Device parameters at the sweetpoint.** We show the $|0\rangle \leftrightarrow |1\rangle$ transition frequency at the sweetpoint for each qubit, the resonance frequencies of all resonators, as well as each element's measured T_1 and T_2^*.[5] Each qubit's coherence is measured at the listed frequency while all other qubits are detuned to 500-700 MHz below. The poor performance of Q_2 at its sweetpoint is likely due to the interference by two-level defects, and Q_5 may be affected as well (see the main text for each qubit's coherence performance at its gate frequency). The resonance frequency of the bus resonator R is noted as its bare frequency ω_{rb}. The coupling strength g_{readout} between each qubit (σ^+ and σ^-) and its own readout resonator ($a^\dagger_{\text{readout}}$ and a_{readout}) is estimated with the interaction Hamiltonian $\hbar g_{\text{readout}}(\sigma^+ + \sigma^-)(a^\dagger_{\text{readout}} + a_{\text{readout}})$ applied in the dispersive limit. The coupling strength g between each qubit and the bus resonator R ($a^\dagger$ and a) is estimated based on the interaction Hamiltonian $\hbar g(\sigma^+ + \sigma^-)(a^\dagger + a)$ via vacuum Rabi oscillations.

SUPPLEMENTARY NOTE 1

Stark shifts and dynamical phases

Here for the one-qubit case, we consider the interaction between the $|1\rangle \leftrightarrow |2\rangle$ transition of one qubit and the resonator with the coupling strength g_{12}. Taking the resonator frequency conditional on the qubit state $|0\rangle$ to be $\omega_{\rm r}$, the resonator frequency associated with the qubit state $|1\rangle$ is $\omega_{\rm r} + 2\lambda$ due to the qubit-state-dependent resonator frequency shift $\lambda = \frac{g_{01}^2}{\omega_{01}-\omega_{\rm rb}}$, where ω_{01} is the qubit $|0\rangle \leftrightarrow |1\rangle$ transition frequency, g_{01} is the coupling strength between the qubit $|0\rangle \leftrightarrow |1\rangle$ transition and the resonator, and $\omega_{\rm rb}$ ($\equiv \omega_{\rm r} + \lambda$) is the resonator's bare frequency (resonator frequency in absence of qubits). Defining the detuning $\varDelta' = \omega_{12} - (\omega_{\rm r} + 2\lambda)$, where ω_{12} is the qubit $|1\rangle \leftrightarrow |2\rangle$ transition frequency. When the qubit is initially in $|1\rangle$, the interaction between the qubit $|1\rangle \leftrightarrow |2\rangle$ transition and the resonator is described by the effective Hamiltonian (setting $\hbar = 1$)

$$H = \omega_{12} |2\rangle \langle 2| + (\omega_{\rm r} + 2\lambda |1\rangle \langle 1|)\, a^\dagger a + g_{12} \left(a |2\rangle \langle 1| + a^\dagger |1\rangle \langle 2| \right), \tag{1}$$

where the energy of the joint state $|1, 0\rangle$ in the notation of $|qubit,\, resonator\rangle$ without coupling and driving is set to be 0. In the subspace $\{|1, 1\rangle, |2, 0\rangle\}$, the dressed states of the coupled qubit-resonator system are

$$\begin{aligned} |\phi_+\rangle &= \cos\frac{\theta}{2} |2, 0\rangle + \sin\frac{\theta}{2} |1, 1\rangle, \\ |\phi_-\rangle &= \sin\frac{\theta}{2} |2, 0\rangle - \cos\frac{\theta}{2} |1, 1\rangle, \end{aligned} \tag{2}$$

where $\tan\theta = 2g_{12}/\varDelta'$. The eigenenergies of these two dressed states are $E_\pm = \omega_{\rm r} + 2\lambda + \left(\varDelta' \pm \sqrt{4g_{12}^2 + \varDelta'^2}\right)/2$. Then the detunings between the drive and the two dressed states are

$$\begin{aligned} \delta_+ &= \delta + \omega_{\rm r} - E_+ = \delta - 2\lambda - \left(\varDelta' + \sqrt{4g_{12}^2 + \varDelta'^2}\right)/2, \\ \delta_- &= \delta + \omega_{\rm r} - E_- = \delta - 2\lambda + \left(-\varDelta' + \sqrt{4g_{12}^2 + \varDelta'^2}\right)/2, \end{aligned} \tag{3}$$

where δ is the frequency difference between the drive and the resonator conditional on the qubit state $|0\rangle$.

Due to the microwave crosstalk on the circuit chip, the qubit is also slightly driven when the drive is intentionally applied to the resonator. To model this case we use a crosstalk

driving strength $\mathit{\Omega}'$ of the qubit $|1\rangle \leftrightarrow |2\rangle$ transition. Under the condition $|\delta_\pm| \gg \mathit{\Omega}$, $\mathit{\Omega}'$, the drive cannot pump the system from the state $|1,0\rangle$ to the dressed states $|\phi_\pm\rangle$, but produces a Stark shift given by

$$\varepsilon = \frac{\left(\mathit{\Omega}' \cos\frac{\theta}{2} + \mathit{\Omega} \sin\frac{\theta}{2}\right)^2}{\delta_+} + \frac{\left(\mathit{\Omega}' \sin\frac{\theta}{2} - \mathit{\Omega} \cos\frac{\theta}{2}\right)^2}{\delta_-}. \tag{4}$$

Assuming $\mathit{\Omega}' = k\mathit{\Omega}$, we have

$$\varepsilon = \mathit{\Omega}^2 \left[\frac{\left(k \cos\frac{\theta}{2} + \sin\frac{\theta}{2}\right)^2}{\delta_+} + \frac{\left(k \sin\frac{\theta}{2} - \cos\frac{\theta}{2}\right)^2}{\delta_-}\right]. \tag{5}$$

In our experiment, k is measured to be ≈ 0.6 at the gate frequency (here k being relatively large is likely due to insufficient crossover grounding wires in our circuit). Due to this energy shift, the system state $|1,0\rangle$ acquires a dynamical phase $\theta_\mathrm{d} = -\varepsilon T$ during the application of the drive.

For a qubit with the anharmonicity and g_{12} both being large enough, θ_d is naturally quenched by tuning $\mathit{\Delta}'$ to 0. For a given device with a limited parameter space accessible, we can still adjust ε by varying $\mathit{\Delta}'$ when other parameters are fixed. When $\varepsilon = 0$, no dynamical phase is accumulated. Here we numerically solve $\varepsilon = 0$ with ε given by Eq. 5 to find the approximate solution, and adjust the qubit frequency accordingly to observe the geometric phase.

Due to the fluctuation in the drive amplitude, the Stark shift deviates from the expected value by

$$\delta\varepsilon \simeq \frac{2\delta\mathit{\Omega}}{\mathit{\Omega}}\varepsilon. \tag{6}$$

Then the correction to the dynamical phase is

$$\delta\phi = -\frac{2\varepsilon}{\mathit{\Omega}} \int_0^T \delta\mathit{\Omega} dt. \tag{7}$$

Suppose that the fluctuation is Gaussian with the correlation function $\langle \delta\mathit{\Omega}(t)\delta\mathit{\Omega}(t+\tau)\rangle = \sigma^2 e^{-\Gamma\tau}$, where σ^2 is the variance and Γ is the noise bandwidth (correlation time $1/\Gamma$). Consequently, the variance of the dynamical phase is given by

$$\begin{aligned} \langle \delta^2\phi \rangle &= \frac{8\sigma^2\varepsilon^2}{\mathit{\Omega}^2} \left(\frac{T}{\Gamma} + \frac{e^{-\Gamma T} - 1}{\Gamma^2}\right) \\ &= \theta_\mathrm{d}^2 \frac{8\sigma^2}{\mathit{\Omega}^2} \left(\frac{1}{\Gamma T} + \frac{e^{-\Gamma T} - 1}{\Gamma^2 T^2}\right). \end{aligned} \tag{8}$$

This implies that the mean square error of the dynamical phase is proportional to the dynamical phase itself. For the slow fluctuation with $\Gamma T \ll 1$, Eq. 8 reduces to $\langle \delta^2 \phi \rangle \simeq 4\theta_{\rm d}^2 \sigma^2/\Omega^2$.

For the implementation of the geometric two-qubit gate, when only one qubit is in $|1\rangle$, the system dynamics reduces to the above-mentioned single-qubit case as the other qubit in $|0\rangle$ is not affected by the drive. When both qubits are in $|1\rangle$ the resonator frequency is $\omega_{\rm r} + 2\lambda_1 + 2\lambda_2$, where $\lambda_j = \frac{g_{j,01}^2}{\omega_{j,01} - \omega_{\rm rb}}$ and $\omega_{\rm rb} \equiv \omega_{\rm r} + \lambda_1 + \lambda_2$, with $g_{j,01}$ being the coupling strength between the $|0\rangle \leftrightarrow |1\rangle$ transition of the j-th qubit and the resonator. In this case the detuning between the $|1\rangle \leftrightarrow |2\rangle$ transition of the j-th qubit and the resonator is $\Delta_j' = \omega_{j,12} - (\omega_{\rm r} + 2\lambda_1 + 2\lambda_2)$, where $\omega_{j,12}$ is the $|1\rangle \leftrightarrow |2\rangle$ transition frequency of the j-th qubit. In the basis $\{|21,0\rangle\,,\ |12,0\rangle\,,\ |11,1\rangle\}$, where c, d, and e in the notation $|cd, e\rangle$ denote the excitation numbers of the 1st qubit, the 2nd qubit, and the resonator, respectively, the dressed states of the coupled qubit-resonator system are

$$|\phi_k\rangle = \mathcal{N}_k \left(|21,0\rangle + \frac{E_k\left(E_k - \Delta_1'\right) - g_{1,12}^2}{g_{1,12}g_{2,12}} |12,0\rangle + \frac{E_k - \Delta_1'}{g_{1,12}} |11,1\rangle \right) \ \text{for}\, k = 1,\ 2,\ \text{and}\, 3, \tag{9}$$

where $\mathcal{N}_k = \left[1 + \left(\frac{E_k\left(E_k - \Delta_1'\right) - g_{1,12}^2}{g_{1,12}g_{2,12}} \right)^2 + \left(\frac{E_k - \Delta_1'}{g_{1,12}} \right)^2 \right]^{-1/2}$, and E_k are the eigenenergies given by

$$\begin{aligned} E_1 &= \left[-\frac{q}{2} + \lambda \right]^{1/3} + \left[-\frac{q}{2} - \lambda \right]^{1/3} + \frac{\Delta_1' + \Delta_2'}{3}, \\ E_2 &= \eta \left[-\frac{q}{2} + \lambda \right]^{1/3} + \eta^2 \left[-\frac{q}{2} - \lambda \right]^{1/3} + \frac{\Delta_1' + \Delta_2'}{3}, \\ E_3 &= \eta^2 \left[-\frac{q}{2} + \lambda \right]^{1/3} + \eta \left[-\frac{q}{2} - \lambda \right]^{1/3} + \frac{\Delta_1' + \Delta_2'}{3}, \end{aligned} \tag{10}$$

with

$$\begin{aligned} \lambda &= \sqrt{\left(\frac{q}{2}\right)^2 + \left(\frac{p}{3}\right)^3}, \\ p &= \frac{-3\left(g_{1,12}^2 + g_{2,12}^2 - \Delta_1'\Delta_2'\right) - \left(\Delta_1' + \Delta_2'\right)^2}{3}, \\ q &= \left(g_{1,12}^2 \Delta_2' + g_{2,12}^2 \Delta_1' - \Delta_1'\Delta_2'\right) - \frac{1}{3}\left(\Delta_1' + \Delta_2'\right)\left(g_{1,12}^2 + g_{2,12}^2 - \Delta_1'\Delta_2'\right) + \frac{2}{27}\left(\Delta_1' + \Delta_2'\right)^3, \\ \eta &= \left(-1 + \sqrt{3}i\right)/2, \end{aligned} \tag{11}$$

where the energy of the state $|11,0\rangle$ without coupling and driving is set to be 0. Setting the frequency difference between the drive and the resonator conditional on the two-qubit state

$|00\rangle$ to be δ, the energy differences between the drive and the dressed states are

$$\delta_k = \delta + \omega_{\rm r} - E_k. \tag{12}$$

Under the condition $|\delta_k| \gg \varOmega$, $\varOmega'_1$, $\varOmega'_2$, where $\varOmega$ is the coupling between the drive and the resonator and $\varOmega'_j$ the coupling between the drive and the $|1\rangle \leftrightarrow |2\rangle$ transition of the j-th qubit, the Stark shift of the state $|11,0\rangle$ due to off-resonantly coupling to these dressed states is

$$\varepsilon' = \sum_{k=1}^{3} \mathcal{N}_k^2 \frac{\left| \varOmega'_1 + \frac{E_k\left(E_k - \varDelta'_1\right) - g_{1,12}^2}{g_{1,12} g_{2,12}} \varOmega'_2 + \frac{E_k - \varDelta'_1}{g_{1,12}} \varOmega \right|^2}{\delta_k}. \tag{13}$$

SUPPLEMENTARY NOTE 2

Device fabrication and parameters

Device fabrication. The five-qubit circuit architecture was designed in a way similar to those outlined previously[1,2], with aluminum bonding-wire crossovers, each about 25 µm in diameter and roughly 1 mm in length, manually applied as many as possible to reduce the impact of parasitic slotline modes. Individual circuit chip was fabricated in a two-step deposition process to minimize contamination: (1) aluminum deposition onto the single-crystal sapphire substrate followed by e-beam lithography and wet etching to define the base wiring including all resonators and control lines; (2) double-angle aluminum deposition onto the e-beam lithography-patterned resist followed by a liftoff process to shape the two-junction superconducting quantum interference device (SQUID). The substrate was pre-heated to above 200°C in the vacuum of the Plassys e-beam evaporator (MEB550) with a background pressure around 5×10^{-8} Torr for more than 2 hours to remove any possible surface defects, and all subsequent depositions of aluminum and the junction oxidation were done in MEB550. Coupling between each qubit and the bus/readout resonator was realized by a fixed-value interdigitated capacitor[1].

Except for rare occasions such as being interfered by two-level defects, the qubit fabricated using the above-mentioned recipe typically demonstrates decent coherence performance at the sweetpoint where the qubit resonant frequency reaches maximum, with the energy relaxation time T_1 and Gaussian dephasing time[5] T_2^* both above 10 µs. The sweetpoint param-

eters for all five qubits on the experimental circuit chip are summarized in Supplementary Table 1.

The impedance-transformed Josephson parametric amplifier (JPA) was fabricated using the conventional multi-layer lithographic recipe, similar to those used for phase qubits and JPAs[1,3]. It was produced in a four-step deposition process on the single-crystal silicon substrate with 500 nm of surface oxide: (1) a layer of 100-nm-thick aluminum was first deposited, followed by e-beam lithography and wet etching to pattern the base wiring; (2) a layer of 250-nm-thick amorphous silicon was coated by plasma enhanced chemical vapor deposition, followed by e-beam lithography and dry etching to define the qubit shunt capacitor, all vias, and all signal transmission line crossovers; (3) after another round of e-beam lithography to pattern the resist, a layer of 160-nm-thick aluminum was deposited followed by a liftoff process to fill the vias for contacting the base wiring and to cap the amorphous silicon dielectrics for finalizing structures such as the capacitor and crossovers, thus completing the top wiring; (4) finally the two-junction SQUID was laid down in a way similar to that in the qubit fabrication procedure except that here the targeting junction resistance is typically 100 times smaller.

Along the signal transmission line of the JPA, the crossover separation is continuously varied, in a manner of the Klopfenstein taper, to transform the environmental characteristic impedance from 50 to 15 Ω, which enables the JPA to yield gains no less than 14 dB and noises near the quantum limit over a bandwidth up to 240 MHz centering around 6.7 GHz, suitable for simultaneously measuring up to six qubits with multiplexing. With this JPA in the measurement setup similar to that described previously[4], the representative measurement fidelities of $|0\rangle$, $|1\rangle$, and $|2\rangle$ for, e.g., Q_1, are 0.96, 0.85, and 0.74, respectively. The typical microwave readout data plotted in the I-Q plane are shown in Supplementary Fig. 1.

Gate and readout frequencies. As pointed out in the main text, during the gate operation it is desired that all qubit ω_{12}s be close to the bus resonator, while all qubit ω_{01}s be away from the bus resonator as much as possible and differ from each other by more than the dispersive coupling strength. To optimize the gate fidelity, we need to carefully address each qubit, with the capability of dynamically biasing its resonance frequency during the pulse sequences of the multiqubit controlled-phase gates. We choose two frequencies for each qubit involved in the gate when necessary: One is for gate operation and the other one is

for readout.

The gate frequencies of these qubits are close to each other since their ω_{12}s are close to the bus resonator and their anharmonicities are similar. But their readout frequencies, if available, are separated more for minimizing the qubit interaction during readout. We also perform single-qubit gates at the readout frequencies when needed, including the tomography and phase compensation rotations.

The gate frequency of each qubit is about 200 to 300 MHz lower than its sweetpoint (maximum) frequency. Within this range of spectrum, the anharmonicity of each qubit, defined as $\omega_{01}/2\pi - \omega_{12}/2\pi$, is around 250 MHz, and T_1 remains approximately constant except for a few spots as interfered by two-level defects (T_1 of Q_5 is above 10 µs at its gate frequency). However, due to enhanced flux noise at lower frequencies, T_2^*s of these qubits all drop significantly at their gate frequencies, measured to be in the range of 2 to 5 µs. We note that the T_2 values used in the master equation simulation are typically much longer than the T_2^* values due to the $1/f$ nature of the noise power spectrum.

SUPPLEMENTARY NOTE 3

Geometric two-qubit CZ gate

For implementation of the two-qubit CZ gate, we arrange the $|0\rangle \leftrightarrow |1\rangle$ transition frequencies of Q_1 and Q_5 to be blue-detuned from the resonator frequency $\omega_r/2\pi$ by 264 MHz and 285 MHz, respectively. Because these detunings are much larger than the corresponding qubit-resonator couplings, the qubits cannot directly exchange excitation with the resonator. Furthermore, the difference between these two detunings is much larger than the dispersive coupling strength, so that the qubits cannot exchange excitation through virtual photon process. With this arrangement and the qubit anharmonicities, the $|1\rangle \leftrightarrow |2\rangle$ transition frequencies of Q_1 and Q_5 are blue-detuned from $\omega_r/2\pi$ by 19 MHz and 41 MHz, respectively. These small detunings ensure that the $|1\rangle \leftrightarrow |2\rangle$ transitions strongly couple to the resonator, and the energy levels of the resulting qubit-resonator dressed states are significantly shifted compared to the corresponding bare states with one photon in the resonator. As a result, the drive cannot pump photons into the resonator when at least one qubit is in the state $|1\rangle$.

We trace the resonator photon number evolution under the external drive with $\Omega/2\pi = 2$ MHz to verify the above argument. The measurement starts with preparing Q_1 and Q_5 in one of the two-qubit computational states, which is followed by tuning the $|1\rangle \leftrightarrow |2\rangle$ transitions of both qubits on near resonance with the resonator. Then the external drive is applied for a variable delay time, following which the resonator state is read out. Supplementary Fig. 2a displays the average photon numbers of the resonator as functions of the delay time conditional on the two-qubit computational states $|00\rangle$, $|01\rangle$, $|10\rangle$, and $|11\rangle$, which are measured by tuning Q_4, initially in its ground state, on resonance with the resonator[6]. As expected, when the qubits are in the state $|00\rangle$, the resonator makes a cyclic evolution, returning to the ground state after a duration of $T = 250$ ns; for the other three computational states, the resonator remains nearly unpopulated.

The geometric phase originates from the cyclic motion of the resonator in the drive frame. To examine the phase acquired by each of the two-qubit computational states during the application of the drive Ω, we perform the Ramsey-type measurements on each qubit (test qubit) with the other one (control qubit) in $|0\rangle$ and $|1\rangle$, respectively (Supplementary Fig. 3). The pulse sequences are similar to that illustrated in Fig. 3a of the main text: For example, the Ramsey-type measurement on Q_5 conditional on Q_1 in $|0\rangle$ (see the bottom-left panel in Supplementary Fig. 3) starts with initializing Q_1 in $|0\rangle$ and Q_5 in $(|0\rangle - i|1\rangle)/\sqrt{2}$ with an $X_{\pi/2}$ gate, which is followed by tuning the $|1\rangle \leftrightarrow |2\rangle$ transitions of both qubits on near resonance with the resonator; then the external drive with a variable strength Ω and a fixed duration of $T = 250$ ns is applied to perform the geometric gate, following which Q_5 is tuned to its readout frequency, where a single-qubit rotation is applied to compensate for the dynamical phase incurred during the frequency change; a $\theta_{\pi/2}$ rotation is subsequently applied before measuring the $|1\rangle$-state probability of Q_5. Here the $\theta_{\pi/2}$ gate rotates the qubit by an angle of $\pi/2$ around the axis with a θ-angle to the x axis in the xy plane, and θ is varied. As expected, when the control qubit is in $|0\rangle$, the geometric phase dominates the total phase difference between the states of $|0\rangle$ and $|1\rangle$ of the test qubit; when the control qubit is in $|1\rangle$, a small dynamical phase is observed.

Based on the geometric phase we construct the two-qubit CZ gate. In addition to using quantum process tomography for characterization as done in the main text, we also examine this CZ gate using interleaved randomized benchmarking (RB), where we insert the CZ gate between random gates from the one- and two-qubit Clifford groups. From the data shown

in Supplementary Fig. 4 we obtain a CZ gate fidelity of 0.939 ± 0.011.

SUPPLEMENTARY NOTE 4
Geometric three-qubit CCZ gate

The CCZ gate is applied on Q_1, Q_3, and Q_5, whose $|0\rangle \leftrightarrow |1\rangle$ transition frequencies are blue-detuned from the resonator frequency $\omega_r/2\pi$ by 268 MHz, 249 MHz, and 285 MHz, respectively, and the $|1\rangle \leftrightarrow |2\rangle$ transition frequencies are blue-detuned from $\omega_r/2\pi$ by 23 MHz, 4 MHz, and 41 MHz. Then the strong couplings to the qubit $|1\rangle \leftrightarrow |2\rangle$ transitions freeze the resonator's evolution when at least one qubit is in the state $|1\rangle$. The pulse sequence for realizing and characterizing the CCZ gate is shown in the left panel of Supplementary Fig. 5, in which the resonance frequencies of Q_1 and Q_5 are dynamically biased for turning on and off the geometric gate.

To examine the conditional phase shift, we perform the Ramsey-type test on each qubit with the other two qubits, acting as the control qubits, prepared in different computational states, which is similar to the Ramsey experiment being carried out for the two-qubit case. The measured probabilities of the test qubit in state $|1\rangle$ after the second $\pi/2$ rotation, as functions of θ and Ω^2, are shown in the right panel of Supplementary Fig. 5. The open circles denote the measured relative phase between $|1\rangle$ and $|0\rangle$ accumulated during the application of the drive, while the solid lines describe the geometric phase calculated as $-2\pi(\Omega/\delta)^2$. The results show that the phase obtained by $|000\rangle$, which is of mainly geometric origin in the drive frame, is much larger than those acquired by other computational states that are of dynamical origin, and which are one of the main sources of gate error.

SUPPLEMENTARY REFERENCES

[1] Lucero, E. *et al.* Computing prime factors with a Josephson phase qubit quantum processor. *Nature Physics* **8**, 719-723 (2012).

[2] Barends, R. *et al.* Coherent Josephson qubit suitable for scalable quantum integrated circuits. *Phys. Rev. Lett.* **111**, 080502 (2013).

[3] Mutus, J.Y. *et al.* Strong environmental coupling in a Josephson parametric amplifier. *Appl. Phys. Lett.* **104**, 263513 (2014).

[4]Kelly, J. *et al.* State preservation by repetitive error detection in a superconducting quantum circuit. *Nature* **519**, 66-69 (2015).

[5]Sank, D. *et al.* Flux noise probed with real time qubit tomography in a Josephson phase qubit. *Phys. Rev. Lett* **109**, 067001 (2012).

[6]Hofheinz, M. *et al.* Synthesizing arbitrary quantum states in a superconducting resonator. *Nature* **459**, 546549 (2009).
